# Targeting TNF-α and NF-κB Activation by Bee Venom: Role in Suppressing Adjuvant Induced Arthritis and Methotrexate Hepatotoxicity in Rats

**DOI:** 10.1371/journal.pone.0079284

**Published:** 2013-11-20

**Authors:** Samar F. Darwish, Wesam M. El-Bakly, Hossam M. Arafa, Ebtehal El-Demerdash

**Affiliations:** 1 Pharmacology and Toxicology Department, Faculty of Pharmacy, Future University, Cairo, Egypt; 2 Pharmacology Department, Faculty of Medicine, Ain Shams University, Cairo, Egypt; 3 Pharmacology and Toxicology Department, Faculty of Pharmacy, MTI University, Cairo, Egypt; 4 Pharmacology and Toxicology Department, Faculty of Pharmacy, Ain shams University, Cairo, Egypt; Wayne State University, United States of America

## Abstract

Low dose methotrexate is the cornerstone for the treatment of rheumatoid arthritis. One of its major drawbacks is hepatotoxicity, resulting in poor compliance of therapy. Dissatisfied arthritis patients are likely to seek the option of complementary and alternative medicine such as bee venom. The combination of natural products with modern medicine poses the possibility of potential interaction between the two groups and needs investigation. The present study was aimed to investigate the modulatory effect of bee venom acupuncture on efficacy, toxicity, and pharmacokinetics and tissue disposition of methotrexate. Complete Freund's adjuvant induced arthritic rats were treated for 3 weeks with methotrexate and/or bee venom. Arthritic score, ankle diameter, paw volume and tissue expression of NF-κB and TNF-α were determined to assess anti-arthritic effects, while anti-nociceptive effects were assessed by gait score and thermal hyperalgesia. Methotrexate toxicity was assessed by measuring serum TNF-α, liver enzymes and expression of NF-κB in liver. Combination therapy of bee venom with methotrexate significantly improved arthritic parameters and analgesic effect as compared to methotrexate alone. Bee venom ameliorated serum TNF-α and liver enzymes elevations as well as over expression of NF-κB in liver induced by methotrexate. Histological examination supported the results. And for the first time bee venom acupuncture was approved to increase methotrexate bioavailability with a significant decrease in its elimination. Conclusion: bee venom potentiates the anti-arthritic effects of methotrexate, possibly by increasing its bioavailability. Also, it provides a potent anti-nociceptive effect. Furthermore, bee venom protects against methotrexate induced hepatotoxicity mostly due to its inhibitory effect on TNF-α and NF-κB.

## Introduction

Rheumatoid arthritis (RA) is a chronic inflammatory disorder characterized by cellular infiltration and proliferation of synovium, leading to progressive destruction of the joints [1]. Proinflammatory cytokines like interleukin-1 and tumor necrosis factor-alpha (TNF-α) are highly expressed in the rheumatoid joint and play a key role in the pathogenesis of RA [2]. These cytokines stimulate the release of chemokines, metalloproteinases, prostaglandin E2 and cycloxygenase-2 from synoviocytes which promote further inflammation, hyperplasia and cartilage destruction [3]. The nuclear factor kappa-B (NF-κB) is a family of transcription factors, mainly p65 that plays a crucial role in different inflammatory diseases, including RA, leading to cartilage destruction and articular damage [4]. Furthermore, synovial tissues from RA patients show massive number of cells expressing NF-κB at the cartilage-pannus junction [5]. In addition, serum and joint tissue TNF-α is usually elevated in those patients [6], hence the use of TNF-α inhibitors suppresses the disease activity.

Generally low-dose weekly methotrexate is the mainstay treatment of RA [7]. However, Hepatotoxicity is one of its major concerns [8], [9]. Furthermore, the ultimate therapeutic goal in RA treatment is remission or at least low disease activity, which may not always be achieved with methotrexate monotherapy and so combination therapy seems to be better. Recent reports found that most of dissatisfied arthritis patients are likely to seek the option of complementary and alternative medicine [10]. The combination of natural products with modern medicine, poses the possibility of potential interaction between the two groups of substances, and it might be of value if it enhances therapeutic potency and minimize adverse effects. Bee venom (BV) is traditionally used for the treatment of chronic inflammatory diseases such as RA and for relief of pain in oriental medicine. A treatment benefit was observed in RA patients treated weekly with BV acupuncture in different clinical trials [11], [12]. BV suppresses leukocyte migration and TNF-α elevation and reduces cytokine production upon uptake of the antigen by dendritic cells [13], [14]. Other studies suggest that BV induce its anti-inflammatory effect via the direct inhibition of NF-κB [15]. A single study in RA patients showed an additive effect when bee sting used simultaneously with classical oral drugs such as methotrexate, sulfasalazine and meloxicam [16].

Accordingly, the current work was designed to address the effect of concurrent administration of BV with methotrexate in the treatment of adjuvant induced arthritis. And since BV is a well-established hepato-protective agent, its ability to circumvent the hepatocelluler toxicity induced by methotrexate was investigated. Furthermore, the present study was extended to elucidate whether the synergistic anti-arthritic effects, produced by the combination of BV and methotrexate, were preliminary due to changes of the pharmacokinetics and tissue disposition of methotrexate or not.

## Materials and Methods

### Drugs and chemicals

Methotrexate vial was purchased from Orion Pharma Co. (Finland). BV (lyophilized whole venom of *Apis millifera*), Complete Freund's adjuvants (CFA), Acetonitrile HPLC grade, Methanol HPLC grade, Trichloroacetic acid and Tris base (Tris–hydroxymethyl-aminomethane) were purchased from Sigma-Aldrich Co. (USA). BV was dissolved in saline and stored at 2°–8°C. CFA consists of a mixture of heat-killed *Mycobacterium tuberculosis* suspended in sterile mineral oil (1 mg/ml). All other chemicals and solvents were of highest grade and commercially available.

### Animals

The study was approved by the ethical guidelines of the Research Ethics Committee of Faculty of Medicine, Ain Shams University (FMASU-REC). FMASU-REC operates under Fedral Wide Assurance No. FWA 00006444. 80 male Wister rats weighing 150–200 g were obtained from Nile Co. for Pharmaceutical and Chemical Industries, Egypt. Rats were housed in an air-conditioned atmosphere, at a temperature of 25°C with alternatively 12 hour light and dark cycles. They were kept on a standard diet and water *ad libitum*. Standard diet pellets contained not less than 20% protein, 5% fiber, 3.5% fat, 6.5% ash and a vitamin mixture. Rats were allowed to have seven days acclimation before any experimentation and the food was placed on the sawdust in the cage to minimize the need for animals to make potentially painful movements to obtain food.

### Experimental design

#### Pharmacodynamic study

To induce arthritis in rats, 0.1 ml CFA was injected subcutaneously into the plantar surface of the left hind paw. Another booster intra-dermal injection of 0.1 ml was given into the root of the tail on the same and on the following day [17]. CFA was used at low concentration of 1 mg/ml due to ethical considerations to minimize animal morbidity and mortality. Fifty rats were divided into five groups (n = 10). A normal group injected only with saline, a control group (CFA induced arthritis), a group with CFA induced arthritis that received methotrexate (0.75 mg/kg/week for 3 weeks, intra-peritoneal) [18], a group with CFA induced arthritis that received BV (0.5 mg/kg 3 times per week for 3 weeks, subcutaneously at Zusanli acupoint) [19], [20], and a group with CFA induced arthritis that received both methotrexate and BV. All treatments started once the symptoms of arthritis appeared on the day after the induction of arthritis, and continued for 21 days. At the end of experiment, blood samples were collected and centrifuged at 1000 g for 10 min. Serum samples were stored at −80°C for analysis. Animal sacrifice was done under sodium phenobarbital anesthesia (150 mg/kg, i.p.), and all efforts were made to minimize suffering.

#### Assessment of arthritis

The progression of CFA induced arthritis was evaluated on days 0, 1, 3, 6, 8, 10, 13, 15, 17 and 21 after adjuvant injection. Arthritic score was used as a semi quantitative parameter of polyarthritis severity through a well-established, widely used scoring system [21]. Paws were examined and graded for severity of erythema and swelling using a 5-point scale: 0 = no signs of inflammation, 1 = swelling and eryhtema of the digit, 2 = moderate swelling and eryhtema, 3 = severe swelling and eryhtema of the limb and 4 = severe swelling, erythema, gross deformity and disability to use the limb. The maximum arthritic score per rat was set at 16 (4 points×4 paws). Ankle diameter was determined by measuring the antero-posterior diameter, using vernier caliper which is accurate to 0.02 mm [22]. Paw volume was measured with a plethysmometer (Type 7140, Ugo Basile, Italy) [23]. Pre-injection values for paw volume and ankle diameter were measured just prior to adjuvant injection for each rat and used as baseline, two measurements were carried out each time for each rat, and the average value was used.

The severity of arthritis pain was measured using gait scoring system as a semi quantitative parameter from 0 to 3: 0 = normal gait, 1 = with slight lameness, 2 = lameness with weight bearing on toes only, 3 = non weight bearing animals [24]. Thermal hyperalgesia response was also assessed using hot-plate (Ugo Basile, model-DS 37). Briefly, animals were placed in a glass cylinder of 24-cm diameter, which was heated and maintained at 50±1°C. The time between placement and shaking or licking of the paws, was recorded as the index of response latency. A cut-off value of 50 sec was used to prevent paw damage. Each animal was tested before administration of drugs in order to obtain baseline. Thermal paw withdrawal latency (PWL) was expressed as a percentage inhibition from day 0 (basal pre-treatment values) and estimated for each rat [25]. To avoid bias, investigators performing all previous assessments and measurements were blinded to treatment group assignments.

### Immunohistochemical detection of tissue NF-κB p65 and TNF-α

Paraffin embedded paw tissue sections of 3 micron thickness were rehydrated first in xylene and then in graded ethanol solutions. The slides were then blocked with 1% bovine serum albumin in tris buffered saline or phosphate buffered saline (PH 7.4) for 2 h as appropriate. The sections were then immune-stained with one of the following primary antibodies; rabbit polyclonal IgG to rat NF-κB p65 (Santa cruz Biotech, inc., USA) at a concentration of 1 µg/ml in tris buffered saline, or rabbit polyclonal IgG to rat TNF-α primary antibody (Abd serotec, MorphoSys Ltd., UK) at a concentration of 1 mg/ml in phosphate buffered saline. After washing the slides with the appropriate buffer, the sections were incubated with goat anti-rabbit IgG secondary antibody. Sections were then washed again and incubated for 5–10 min in a solution of 0.02% di-amino-benzidine containing 0.01% H_2_O_2_. Counter staining was performed using hematoxylin, and the slides were visualized under a light microscope. The quantification of TNF-α and NF-κB staining was performed by using Leica MDLSD image analysis software. It was represented as the optical density of stained sections per field.

#### Histopathology of hind paw

Paw from different groups were excised, fixed in 10% formalin, decalcified in formic acid, embedded in paraffin blocks from which 5 micron-thick sections were obtained. Sections were stained with hematoxylin and eosin dye, and then evaluated under light microscope [26].

#### Assessment of methotrexate toxicity

Serum alanine aminotransferase (ALT) and aspartate aminotransferase (AST) were determined calorimetrically [27]. Serum TNF-α was estimated using Quantikine® ELISA kit (R&D Systems, USA) according to the manufacturer's instructions. After animals were sacrificed, tissues of liver were fixed in 10% formalin solution, cleared in xylene, embedded in paraffin blocks from which 5 micron-thick sections were obtained. Sections were stained with hematoxylin and eosin dye and examined for pathological changes under light microscope by a pathologist blind to the specimens. [28]. In addition, liver sections were immuno-stained with the primary antibody; rabbit polyclonal IgG to rat NF-κB p65 (Santa cruz Biotech, inc., USA).

#### Pharmacokinetic study

Thirty male Wister rats were classified into two groups (n = 15). Animals were given either normal saline or BV (0.5 mg/kg 3 times/w, s.c. at Zusanli acupoint) for 3 weeks. Then on day 22, all animals were injected with a single dose of methotrexate (0.75 mg/kg, i.p). Immediately after methotrexate administration, blood samples were withdrawn over 24 h (5 and 30 min, 1, 2, 4, 7 and 24 h) by puncturing retro orbital sinus under ether anesthesia which doesn't affect methotrexate pharmacokinetics [29]. Plasma samples were immediately obtained by centrifugation at 1000 g for 10 min. The withdrawal of synovial fluid was performed by injection of 200 µL of 0.9% NaCl intra-articularly into each knee and aspirated with a syringe, after which the synovial membrane was excised with the aid of medical magnifying glass [30]. Finally, tissues of lung, heart, liver, spleen and kidney were isolated, washed with ice-cold saline and weighted. Plasma, synovial fluid and tissue samples were stored at −80°C till analysis.

#### Assessment of plasma and tissue kinetics after single methotrexate dose

High performance liquid chromatography (HPLC) was used for determination of methotrexate levels in plasma and tissue homogenates of rats [31]. A stock standard solution of methotrexate (90.8 µg/ml) was prepared using Tris-HCl buffer (pH 8): acetonitrile, 9:1, respectively to provide adequate stability and solubility [32]. Stock solutions of internal standard (p-amino-acetophenone) were also prepared in the same solvent at a concentration of 50 µg/ml. Further dilution of methotrexate stock solution was done to prepare different concentrations of methotrexate (90.8, 45.4, 22.7, 11.4, 5.7, 2.8, 1.4 µg/ml) for the construction of the calibration curve. Isolated tissues were homogenized in phosphate-Tris buffer (0.1M dihydrogen phosphate and 0.01 M Tris; pH 5.7) to obtain 15% (w/v) tissue homogenate, then centrifuged at 1000 g for 10 minutes. The aliquots were aspirated and transferred to Eppendorff's tubes just prior to analysis. To each 200 µl of test (plasma, synovial fluid or aliquots of tissue homogenate) or standard sample, 20 µl of stock solutions of internal standard was added and after complete mixing, 40 µl of trichloroacetic acid (2 M in ethanol) was added to precipitate proteins, and vortex mixed for 2 minutes, then centrifuged at 1000 g for 15 minutes. 20 µl aliquots of the supernatant were directly injected into the chromatography column. Each sample was analyzed in duplicate. Standard solutions were freshly prepared, tubes were covered with foil and analyzed immediately to decrease the degradation of methotrexate because of its light sensitivity. HPLC analysis was performed using Beckman apparatus (system Gold, dual pump and kanauer injector with 20 ul loop, and variable UV detector), and chromatographic column was a 250×4.6 (i.d)-millimeter, lichrosorb C18 with 5-micron particles (Phenomenex, ID. USA). The mobile phase consisted of phosphate –Tris buffer (0.1M dihydrogen phosphate and 0.01 M Tris; pH 5.7): methanol: acetonitrile, with the ratio of 82:11:7, respectively. The flow rate was 1.8 ml/minute. Ultraviolet detection was done at 313 nm at room temperature (25°C).

#### Data analysis and statistical procedures

To analyze two sets of data, unpaired Student's t-test was used. To analyze more than two sets of data, one way or repeated mesures analysis of variance (ANOVA) was used followed by Tukey-Kramer test for multiple comparisons as appropriate. To analyze non-parametric data, Kruskal-Wallis test was used followed by Dunn's test for multiple comparisons. Analysis of data and presentation of graphs were performed using GraphPad Prism software version 5 (ISI® software, USA). Pharmacokinetic analysis was evaluated by standard non-compartmental techniques using the program Kinetica version 5.0 (Thermo Fischer Scientific, Waltham, MA, USA).

## Results

### Anti-arthritic effects

**Figure 1:**
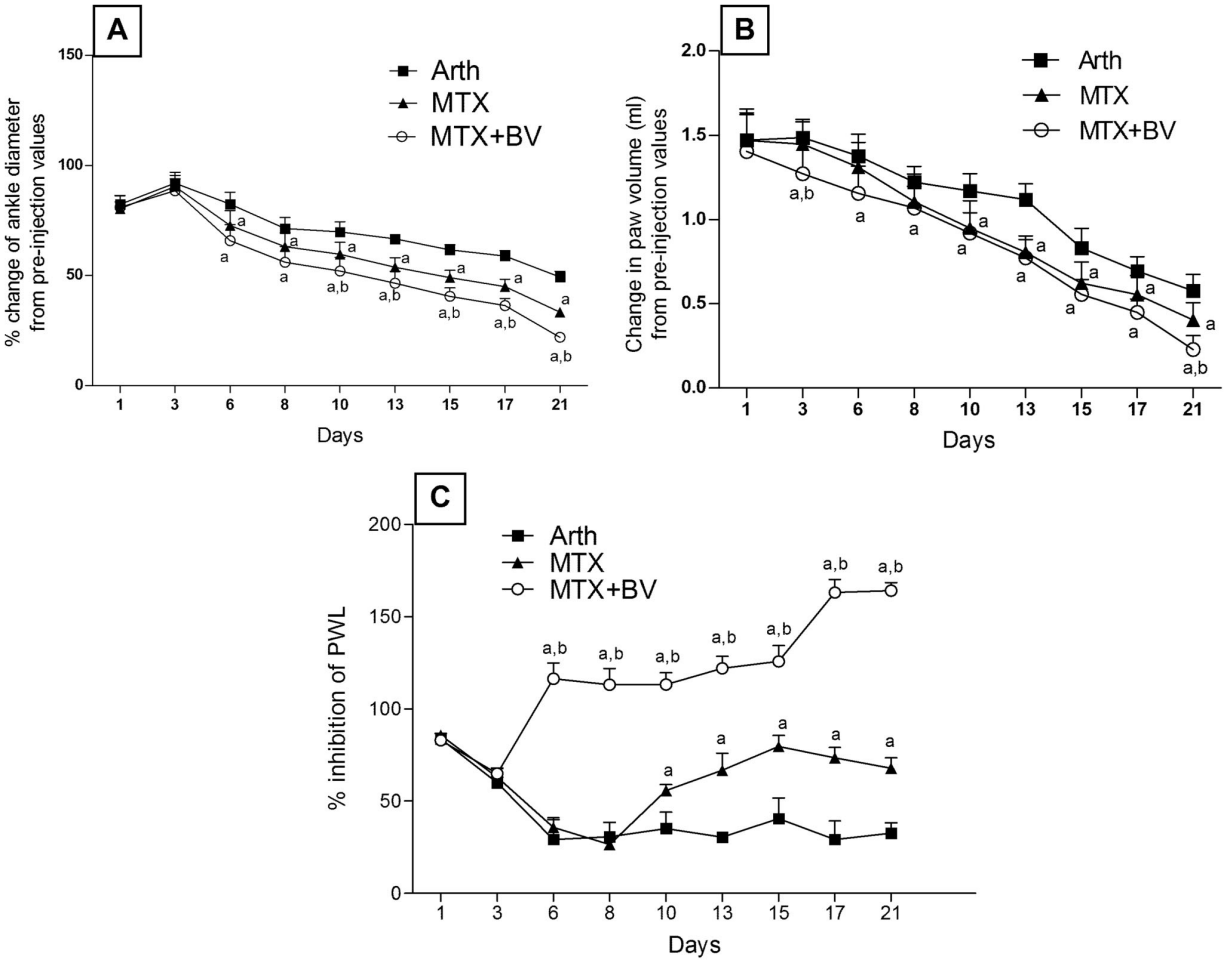
Effect of methotrexate and/or BV on ankle diameter, paw volume and paw withdrawal latency. Panel A: percentage change in ankle diameter, panel B: paw volume changes and panel C: percentage inhibition of paw withdrawal latency (PWL) from pre-injection values in adjuvant induced arthritic rats over a period of 21 days. Data are represented as mean ± SD. a or b: significantly different from the corresponding Arth or MTX group, respectively at P<0.05 using repeated measures of ANOVA followed by Tukey-Kramer Multiple Comparison Test.

**Figure 2:**
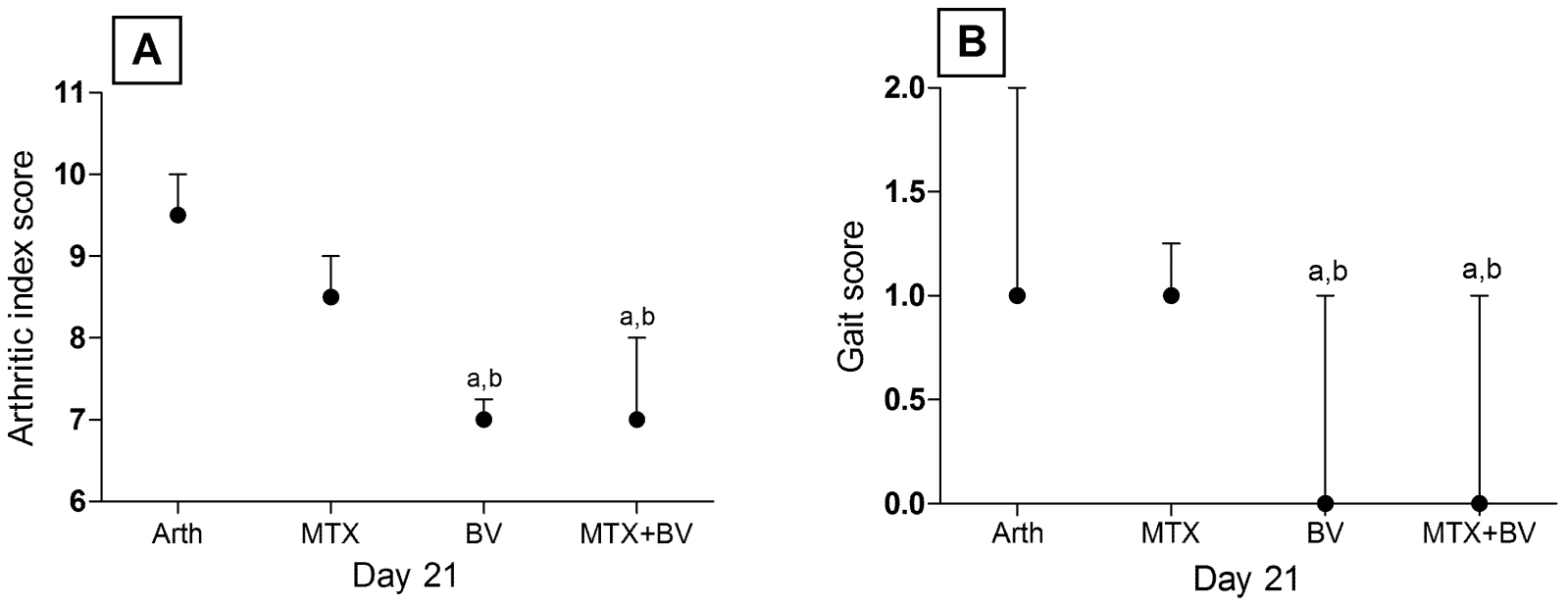
Effect of methotrexate and/or BV on arthritic index and gait score. Panel A: arthritic index score and panel B: gait score on day 21 in adjuvant induced arthritis. Data are represented as medians and interquartile ranges (n = 10). a or b: significantly different from the corresponding Arth or MTX group, respectively at P<0.05 using Kruskal-Wallis test followed by Dunn's Multiple Comparison Test.

Twenty-four hours after injection of CFA, all of arthritic treated or non-treated rats showed a significant arthritis and hyperalgesia as compared to normal control group manifested as significant increase in all arthritic parameters. Methotrexate treatment alone significantly (p<0.05) ameliorated the changes in the ipsilateral ankle diameter, paw volume as compared to the change in arthritic non-treated rats. Concurrent treatment with methotrexate and BV had showed earlier anti-edematous effect evidenced by further significant decrease in ankle diameter and paw volume till reaching 66 and 57%, respectively on day 21 as compared to the change in methotrexate treated rats (**Fig. 1A and 1B**). Concerning the arthritic index, concurrent treatment of arthritic rats with methotrexate and BV showed a significant improvement in arthritis severity at the end of the experiment as compared to arthritic non treated rats and rats treated with methotrexate alone, while methotrexate treatment didn't show any improvement from the arthritic non treated rats throughout the experiment (**Fig. 2A**). In the contralateral paw, no significant changes in neither volume nor ankle diameter were observed throughout the experiment in all groups.

Methotrexate analgesic effect was delayed, being first evident on day 10 post induction by showing a significant increase in the PWL and continued to increase till reaching 208% on day 21, as compared to the change in arthritic non treated rats. Combined treatment showed earlier significant increase in PWL starting from day 6 and continued to increase till reaching 500 and 242% on day 21, as compared to the change in arthritic non treated rats as well as rats treated with methtrexate alone, respectively. It is worth mentioning that neither arthritic non-treated rats nor rats treated with methotrexate alone returned to their normal values till the end of the experiment, while rats concurrently treated with BV and methotrexate returned to their normal values and succeeded them by 164% at the end of the experiment (**Fig. 1C**). Furthermore, treatment with BV alone and combined treatment of methotrexate with BV showed a significant decrease in gait score throughout the experiment and on day 21, combined treatment with BV showed further significant decrease in gait score as compared to rats treated with methotrexate alone (**Fig. 2B**).

### NF-κB p65 and TNF- α expression in hind paw

**Figure 3:**
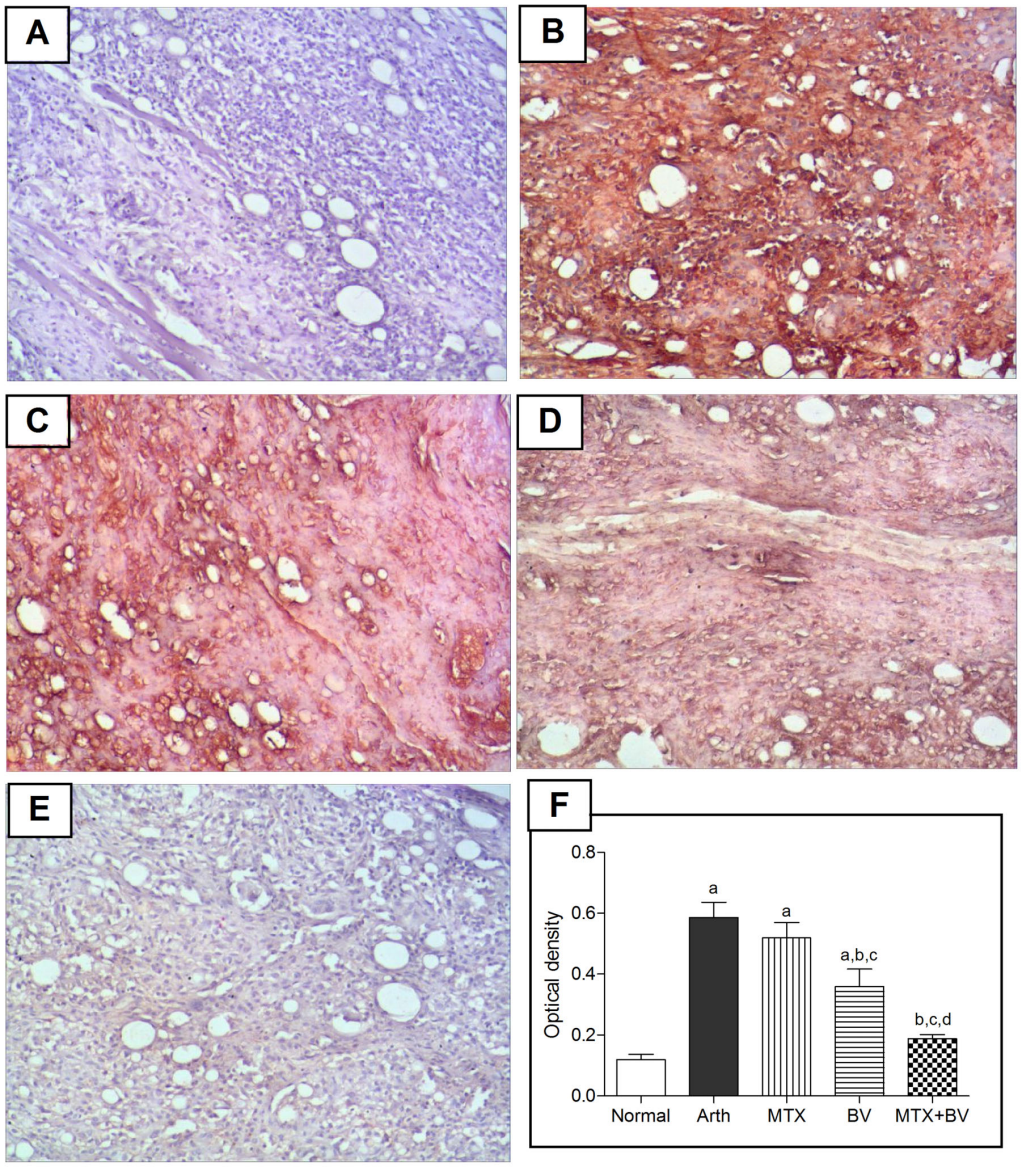
Methotrexate and/or BV effect on Tissue necrosis factor-alpha expression in synovial membrane of hind paw. Immunohistochemical staining (×100) of paw sections of A: normal rat shows almost negative immunostaining for TNF-α, B: arthritic non-treated rat shows massive immunostaining, C: MTX treated rat shows moderate immunostaining, D: BV treated rat shows mild immunostaining and E: concurrently treated rat with MTX and BV shows minimal TNF-α expression. F: mean optical density of synovial membrane stained with TNF-α immunostaining in different studied groups. Data are represented as mean ± SD (n = 10). a, b, c or d: significantly different from the corresponding Normal, Arth, MTX or BV group respectively at P<0.05 using one-way ANOVA followed by Tukey-Kramer Multiple comparison test.

**Figure 4:**
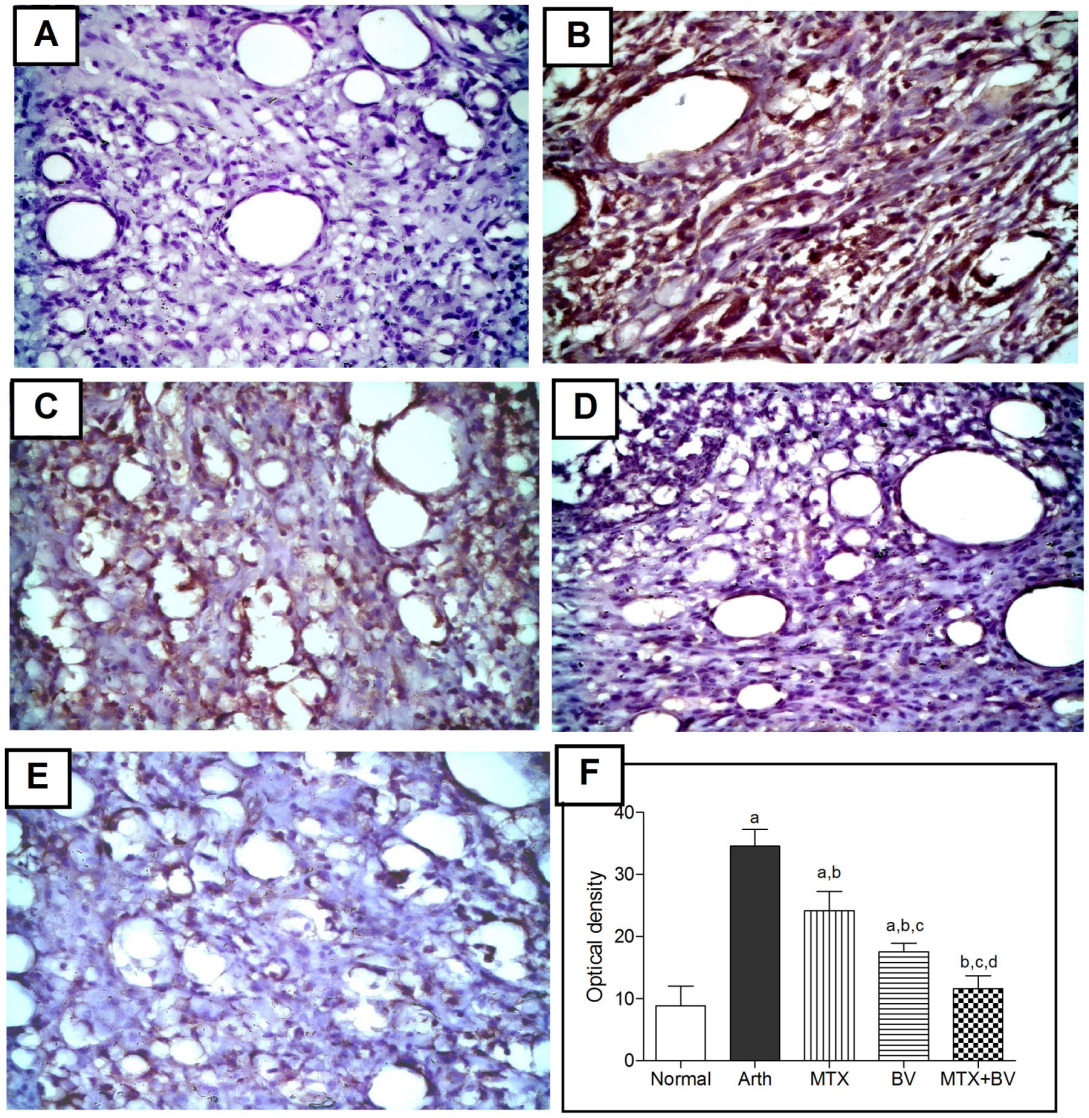
Methotrexate and/or BV effect on NF-κB (p65) expression in synovial membrane of hind paw. Immunohistochemical staining (×400) of paw sections of A: normal rat shows almost negative immunostaining, B: arthritic non-treated rat shows massive immunostaining, C: MTX treated rat shows moderate immunostaining, D: BV treated rat shows mild immunostaining, E: concurrently treated rats with MTX and BV shows minimal NF-κB p65 expression. F: mean optical density of synovial membrane stained with NF-κB p65 immunostaining in different studied groups. Data are represented as mean ± SD (n = 10). a, b, c or d: significantly different from the corresponding Normal, Arth, MTX or BV group respectively at P<0.05 using one-way ANOVA followed by Tukey-Kramer Multiple comparison test.

Immunohistochemical analysis of TNF-α and NF-κB revealed that normal rats showed almost negative immunostaining for both (**Fig. 3A** **and** **4A**). Arthritic non-treated rats exhibited a significant increase in expression of both TNF-α content and the activated subunit of NF-κB (p65) in hind paw tissues by 392 and 290%, respectively, as compared to normal group (**Fig. 3F** **and** **4F**), which was evident from the intense brown staining. Treatment of animals with methotrexate alone showed moderate immunostaining (**Fig. 3C** **and** **4C**), and reduced the optical density significantly in NF-κB by 30% but the decrease in TNF-α wasn't statistically significant as compared to arthritic non-treated rats (**Fig. 4F** **and** **3F**). However, concurrent administration of BV with methotrexate significantly reduced the expression of both TNF-α and NF-κB similar to that in normal rats (**Fig. 3E** **and** **4E**), and induced further significant decrease in optical density by 64 and 66%, respectively, as compared to group treated with methotrexate alone (**Fig. 3F** **and** **4F**).

### Histolopathology of hind paw

**Figure 5:**
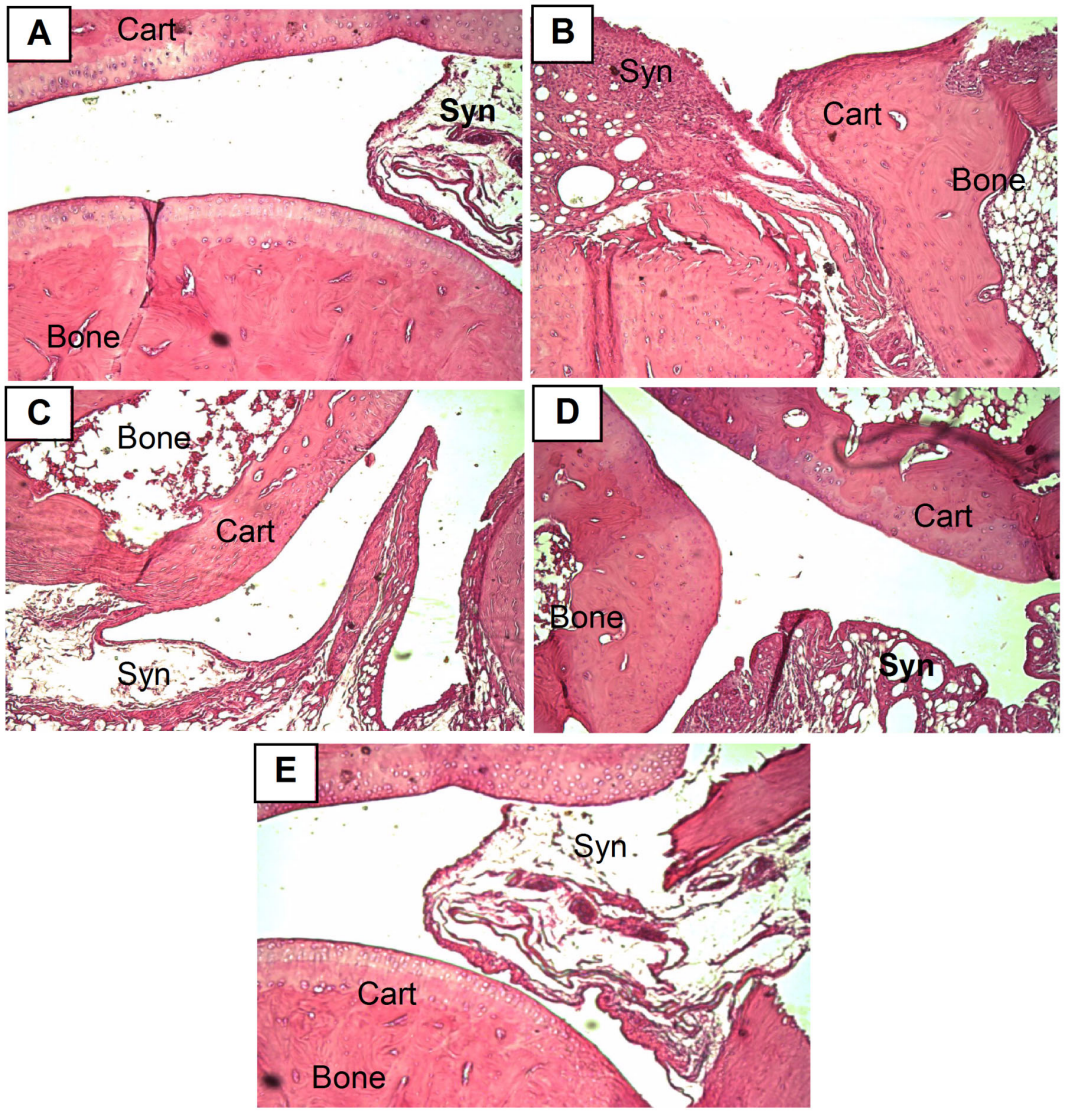
Photomicrographs of hind paw sections stained by H&E (×100). A: paw section from normal rat shows normal appearance of synovial membrane (syn), cartilage (cart) and bone. B: paw section from arthritic non-treated rat shows extensively expanding synovial membrane with pannus formation. C: paw section from arthritic rat treated with MTX shows moderate infiltration with pannus formation. D: paw section from arthritic rat treated with BV alone shows normal synovial membrane with mild infiltration. E: paw section from arthritic rat concurrently treated with MTX and BV shows intact histological structure of cartilage, bone and synovial membrane.

Arthritic non-treated rats showed inflammatory cell infiltration, synovial hyperplasia with multiple cartilage degeneration and pannus formation. Methotrexate treatment alone showed moderate changes. Interestingly, concurrent administration of BV with methotrexate ameliorated any histopathological alterations in synovial membrane, cartilage or bone (**Fig. 5**).

### Methotrexate toxicity

Estimation of liver enzymes showed that CFA injection induced a significant increase in serum ALT and AST levels as compared to normal control group. Methotrexate treatment alone induced further significant increase in both ALT and AST serum levels as compared to arthritic non-treated rats by 52 and 44%, respectively. Concurrent treatment of BV with methotrexate induced a significant decrease in both ALT and AST enzymes as compared to methotrexate treated group almost to their normal levels (**Table 1**).

**Table 1:** Methotrexate and/or bee venom effect on serum AST, ALT and TNF-α in adjuvant arthritic rats.

Groups	ALT conc (U/L)	AST conc (U/L)	Serum TNF-α conc (pg/ml)
Normal	9.8±2.4	21.3±4.2	8.2±2.2
Arth	23.1^a^±0.9	55.5^a^±4.8	23.9^a^±3.6
MTX	35.1^a^^, ^ b±2.6	79.8^a^^, ^ b±4.9	56.6^a^^, ^ b±4.7
BV	13.6^b^^, ^ c±1.9	22.7^b^^, ^ c±5.9	16.8^c^±5.9
MTX+BV	15.1^b^^, ^ c±4.6	31.2^a^^, ^ b ^, ^ c±3.8	36.6^a^^, ^ b ^, ^ c ^, ^ d±5.7

-Data is represented as mean ± SD (n = 10), normal rats (Normal), arthritic non-treated rats (Arth), methotrexate arthritic treated rats (MTX), bee venom arthritic treated rats (BV), concurrently treated arthritic rats with bee venom and methotrexate (MTX+BV).

a, b, c or d : significantly different from the corresponding (Normal), (Arth), (MTX) or (BV) group respectively at P<0.05 using one-way ANOVA followed by Tukey-Kramer multiple comparison test.

Assessment of serum TNF-α concentration revealed that arthritic non-treated rats showed a significant increase by 192%, as compared to normal group. Treatment with methotrexate alone induced further significant increase in serum TNF-α concentration as compared to arthritic non-treated rats. Concurrent administration of BV with methotrexate induced a significant decrease in serum TNF-α concentration by 35%, as compared to methotrexate treated group (**Table 1**).

**Figure 6:**
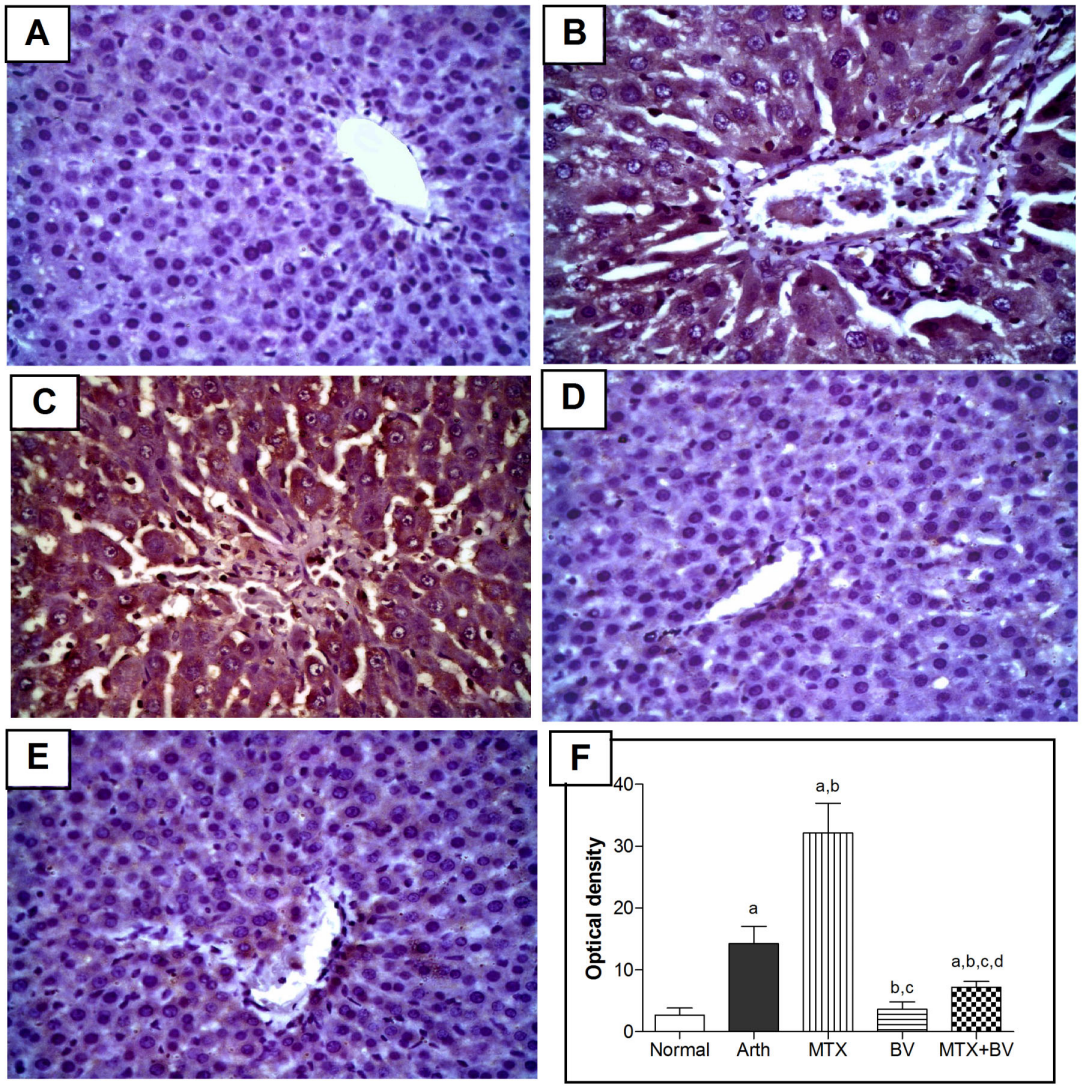
Methotrexate and/or BV effect on NF-κB (p65) expression in liver. Immunohistochemical staining (×400) of liver sections of A: normal rat shows almost negative immunostaining, B: arthritic non-treated rat shows moderate immunostaining, C: MTX treated rat shows intense immunostaining, D: BV treated rat shows mild immunostaining and E: concurrently treated rat with MTX and BV shows minimal NF-κB p65 expression. F: mean optical density of liver tissue sections immunostained with NF-κB p65 in different studied groups. Data are represented as mean ± SD (n = 10). a, b, c or d: significantly different from the corresponding Normal, Arth, MTX or BV group respectively at P<0.05 using one-way ANOVA followed by Tukey-Kramer Multiple comparison test.

Induction of arthritis resulted in a significant increase in the NF-κB p65 expression in the liver tissues by 426%, as compared to normal rats. Methotrexate treatment alone induced a further significant elevation in the expression of NF-κB by 126%, as compared to arthritic non-treated rats, which was evident from the intense brown staining. Concurrent treatment with BV and MTX significantly reduced the expression of NF-κB in liver close to that in normal rats (**Fig. 6**).

**Figure 7:**
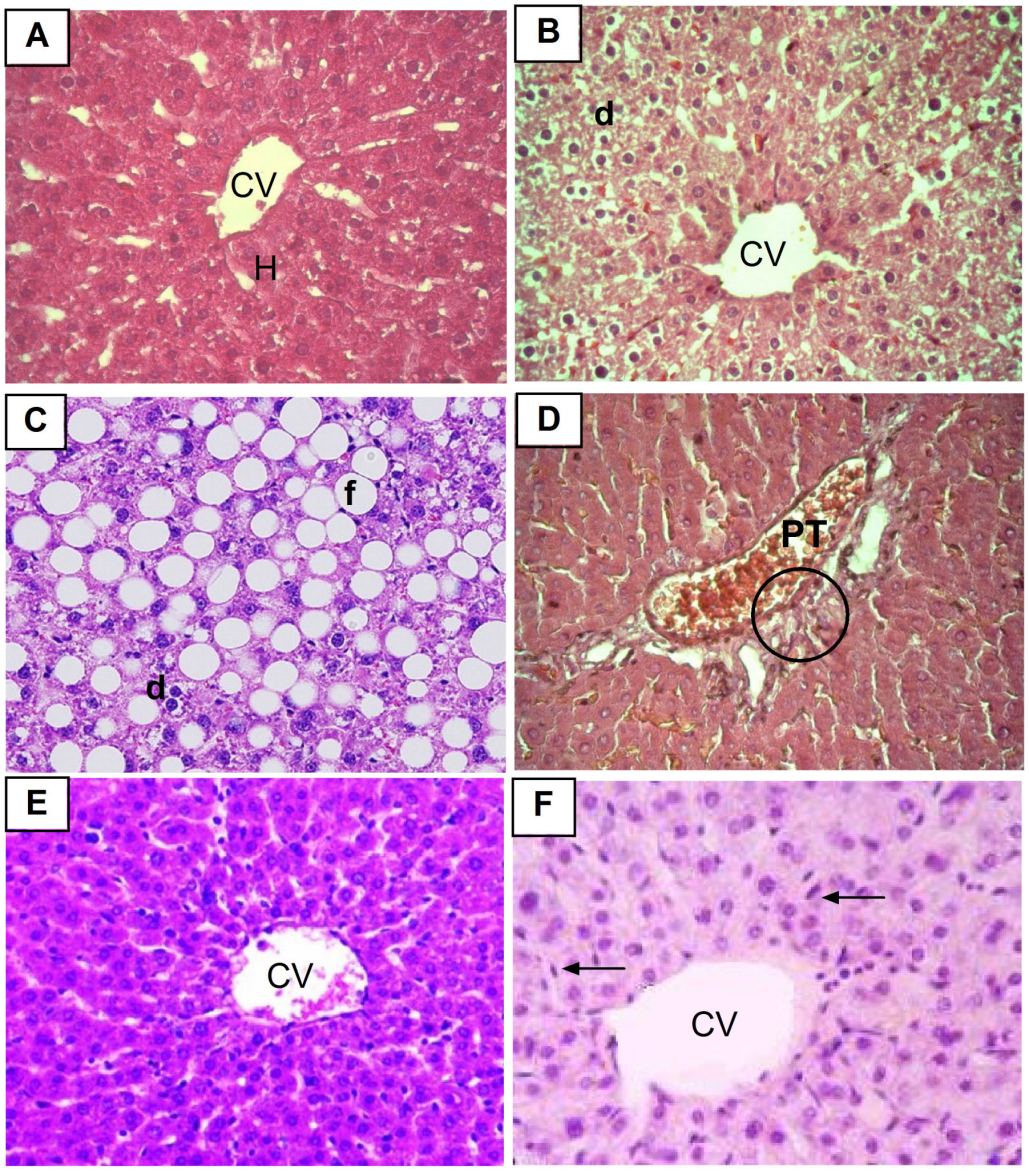
Photomicrographs of liver sections stained by H & E (×400). A: liver section of normal rat shows normal central vein (CV) and surrounding hepatocytes (h). B: liver section of arthritic non-treated rat shows degeneration in hepatocytes (d). C and D: liver sections of arthritic rat treated with MTX alone shows severe fatty change (f), degeneration (d) of hepatocytes, congested portal tract (PT) and lost cell boundaries with distortion of normal architecture (circle). E: liver section of arthritic rat treated with BV alone shows normal hepatic architecture. F: liver section of arthritic rat concurrently treated with MTX and BV showing mild congestion in the central vein (CV) with diffuse kupffer cells proliferation (arrow) in between hepatocytes.

Liver toxicity was further evaluated by histopathological assessment of liver tissue in different groups. Normal rats showed normal histological structure of the central vein with normal surrounding hepatocytes. Arthritic non-treated rats showed mild degenerative changes in hepatocytes. Massive fatty changes, congested portal tract and lost cell boundaries with distortion of normal architecture were observed in liver sections taken from methotrexate treated rats. Concurrent treatment of BV with methotrexate preserved the normal architecture of hepatocytes with mild congestion of central vein (**Fig. 7**).

### Plasma and tissue kinetics after single methotrexate dose

Figure (**8A**) and table (**2**) show that pre-treatment of rats with BV induced a significant changes in pharmacokinetic parameters of methotrexate by showing a significant increase in C_max_, AUC, MRT and t_1/2_ of methotrexate by 209, 258, 105 and 109%, respectively, while there was a significant decrease in K_el_ and CL by 50 and 73%, respectively, as compared to rats injected with methotrexate alone.

**Figure 8:**
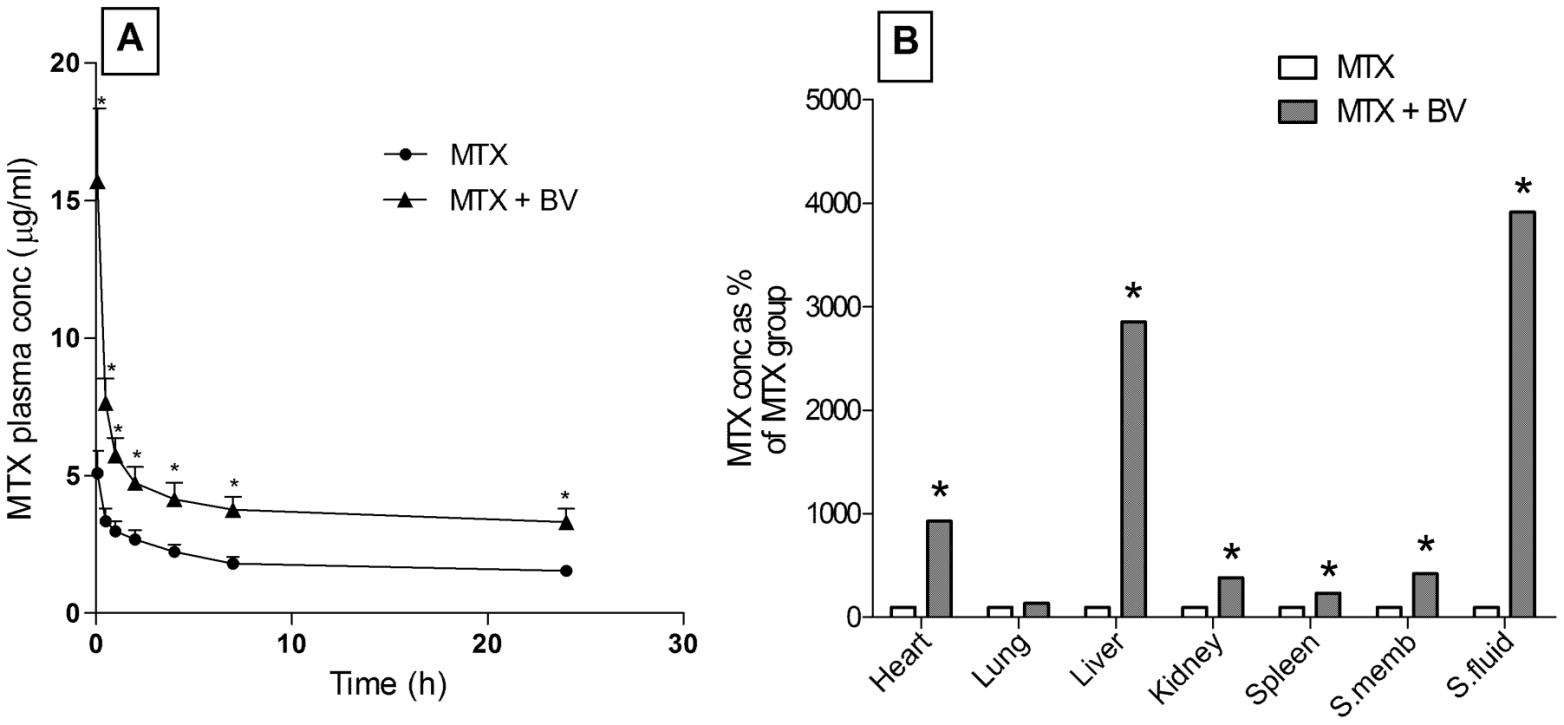
Bee venom effect on plasma concentrations, synovial fluid and tissue concentrations of methotrexate single dose. Panel A: plasma concentrations of methotrexate, panel B: different tissue concentrations of methotrexate. Data are represented as mean ± SD or percentage change of MTX group (n = 15).* means significantly different from the corresponding MTX group at P<0.05 using unpaired student's t-test.

**Table 2:** Effect of bee venom on pharmacokinetic parameters and constants of a single dose of methotrexate.

Parameters	Single MTX	Single MTX+BV
C_max_ (µg/ml)	5.09±0.808	15.71*±2.646
AUC _0-∞_ (µg.h/ml)	110.50±28.520	395.60*±72.240
MRT(h)	44.42±11.960	90.90*±14.390
t_1/2_ (h)	30.59±8.809	63.77*±10.190
K_el_ (hr^−1^)	0.02±0.005	0.01*±0.002
CL (ml/hr)	1.53±0.312	0.42*±0.081

-Data is represented as means ± SD (n = 15), normal rats injected with single dose of methotrexate (Single MTX), normal rats injected with bee venom for 3 weeks followed by single dose of methotrexate (Single MTX+BV),

* means significantly different from the corresponding (single MTX) group at P<0.05 using unpaired student's t-test.

Figure (**8B**) shows that pre-treatment of rats with BV for 3 weeks significantly increased methotrexate concentrations by 832, 2777, 284, 135, 323 and 3825% in heart, liver, kidney, spleen, synovial membrane and synovial fluid, respectively, as compared to group treated with methotrexate alone. However, pre-treatment of BV didn't significantly change methotrexate concentration in lung, as compared to group treated with methotrexate alone.

## Discussion

The present study was designed to address the effect of concurrent use of BV with methotrexate in the treatment of adjuvant arthritis. And since BV is a well-established hepato-protective agent, its ability to circumvent the hepatocelluler toxicity induced by methotrexate was also investigated. Furthermore, the present study was extrapolated to elucidate whether the synergistic anti-arthritic effects, produced by the combination of BV and methotrexate, were preliminary due to changes of the pharmacokinetics and tissue disposition of methotrexate or not.

CFA has been widely used as an experimental model of induced arthritis for evaluation of pharmacological action of anti-arthritic agents. In the present study, it was found that CFA injection induced significant paw edema, high arthritic score and significant elevations of tissue NF-κB and TNF-α, as compared to normal group. These results had been confirmed histologicaly. In addition, the CFA model in this study was characterized by marked increase in the sensitivity of the affected paw to thermal stimulus and hyperalgesia as compared to normal rats. These results were in agreement with the results of other studies that have examined the action of CFA-induced arthritis in rats [33], [34]. Systemic effects of CFA injection was confirmed by elevated serum TNF-α, liver enzymes and liver expression of NF-κB, in addition to the histopathological changes of liver, as compared to normal rats. All these changes confirmed the induction of systemic arthritis with little co-morbidity on animals.

In the present study, CFA model was used to evaluate the anti-arthritic effect of concurrent treatment with methotrexate and BV. Zusanli acupoint was selected in this study, as it has been found that the injection of BV into this acupoint resulted in a significantly greater anti-inflammatory and anti-nociceptive effects on arthritis as compared to BV injection into a more distant non-acupoint. Indeed, apipuncture therapy in Zusanli acupoint had proved its effectiveness in different experimental animal studies [20], [35]–[37], as well as in randomized clinical trials [11], [12], [38]. Our study revealed that, treatment with low dose methotrexate or BV alone significantly produced anti-arthritic effects by improving paw volume and ankle diameter, which supported by histological improvement of hind paw as compared to arthritic non-treated rats, while arthritis scoring was improved by BV treatment only. These results are in agreement with other studies that confirmed the anti-arthritic effects of methotrexate [39], or BV [40]. BV contains a variety of peptides including melittin, apamin, adolapin, and the mast cell degranulating peptide [41]. It also contains enzymes, such as phospholipase A2 (PLA2), biologically active amines (e.g., histamine and epinephrine) and nonpeptide components (including lipids, carbohydrates, minerals and free amino acids) [42].Only a few of these individual components of BV have been tested to date for their possible anti-inflammatory and anti-nociceptive effects. It was reported that adolapin and mast cell degranulating peptides had anti-inflammatory and anti-nociceptive activity [43]. However, it is important to note that these substances are present in very small quantities (1–2%) in dried whole BV. So till now there is no certain component responsible for the antiarthritic effect of BV.

The pathogenesis of RA involves different inflammatory cascades including the transcription factor NF-κB pathway, which plays a pivotal role in the inflammation and hyperalgesia associated with RA [44]. Several lines of recent evidence have also suggested that pro-inflammatory cytokines such TNF-α play a pivotal role in the pathogenesis of RA. TNF-α contributes to synoviocyte proliferation and increases the production of tissue enzymes such as matrix metalloproteinases resulting in cartilage degradation [45]. In the present study, it was found that the marked elevations of NF-κB expression in the synovial membrane of hind paw of non-treated arthritic rats was significantly reduced upon treatment with methotrexate alone, while the reduction of tissue TNF-α expression was not significant, a finding indicates that the anti-inflammatory mechanism of methotrexate, in the current study, is due to inhibition of NF-κB, but not due to inhibition of TNF-α. This finding is in agreement with a previous clinical study which showed that treatment with methotrexate as anti-inflammatory agent didn't statistically reduce TNF-α in tissue [46]. It was suggested that methotrexate clinical effectiveness is mediated through its inhibitory effect on macrophage infiltration, thus affecting interleukins expression, while it doesn't affect T-cell infiltration, which is the main responsible for TNF-α expression.

On the other hand, treatment with BV alone showed significant reduction of both NF-κB and TNF-α expression in synovial membrane of hind paw, as compared to arthritic non-treated group. These results are in agreement with other studies that confirmed the potent anti-inflammatory action of BV through the direct inhibition of NF-κB transcription factor [15], [40] and key inflammatory mediators, such as TNF-α [14], [47], [48]. Moreover, BV had shown a significant analgesic effect as evident by improving PWL and gait score. On the contrary, methotrexate alone showed delayed, mild and non-significant analgesic effect, as compared to arthritic non-treated rats. Different animal models of neuropathic pain had implicated the pivotal role of TNF-α in sensitization at both peripheral and central levels [49]. TNF-α algesic effects are due to sensitizing actions on nociceptive primary afferents and to the up-regulation of other pro-inflammatory and algesic proteins [50]. These findings explain the anti-nociceptive effect of BV and failure of this effect in methotrexate treated group as a result of their different actions on TNF-α.

Concurrent administration of BV with methotrexate would be a promising alternative therapy for the treatment of RA, as the present study showed that this combination improved the arthritic parameters by improving paw volume, ankle diameter and arthritis scoring as compared to methotrexate treated rats. The only previous study that showed a potential synergistic effects of combination therapy of BV and methotrexate in RA treatment was a clinical trial using bee sting not BV acupuncture with oral methotrexate and other anti-rheumatic drugs, and the analgesic effect of such combination was not assessed [16]. In addition, it was found that this combination significantly improved the hyperalgesic parameters by improving PWL and gait score as compared to methotrexate alone. These synergistic effects of concurrent administration of BV with methotrexate might be explained by dynamic or kinetic interactions between the two substances. In the present study, the concurrent administration of BV with methotrexate had significantly decreased the expression of both NF-κB and TNF-α in synovial membrane of hind paw, as compared to methotrexate monotherapy. This finding explained the improvement of both arthritic and hyperalgesic parameters upon concurrent administration of BV with methotrexate as compared to methotrexate monotherapy.

To the best of our knowledge, this is the first time to study the effect of BV on methotrexate pharmacokinetics and tissue disposition; another way to explain the increased methotrexate efficacy. The bioavailability of methotrexate in blood, synovial fluid and synovial membrane were remarkably increased when given to rats pre-treated with BV, as compared to rats injected with methotrexate alone. This finding could explain the ability of BV to potentiate the anti-arthritic effect of methotrexate in adjuvant induced arthritic rats by improving its bioavailability in targeted sites. This was also associated with significant elevation of tissue methotrexate concentrations in different organs. In the present study, methotrexate concentration in liver was greater in rats pre-treated with BV than rats injected with methotrexate alone. So further evaluation is needed to explain why there was increase in liver methotrexate concentration after BV treatment. It could be explained by interaction on the organic anion transporting polypeptides 1A and 1B activity. These transporters have been described previously to be highly expressed in human liver and play an important role in the disposition of methotrexate, primarily by mediating the hepatic uptake of the drug [51], [52].

Different in vivo and in vitro studies found that elevated levels of NF-κB and TNF-α induce apoptosis in hepatocytes, and these findings suggest that both of them play a pivotal role in the pathogenesis of induced acute hepatic injury in different experimental animals [53]–[57]. In the present study, CFA injection induced significant increase in serum TNF-α, ALT and AST enzymes and NF-κB liver expression, in addition to pathological changes in liver, as compared to normal group, which were exaggerated upon methotrexate treatment. These results are consistent with other studies which proved that methotrexate induced hepatotoxicity was associated with elevated serum TNF-α level [58]–[60]. Despite the increased concentration of methotrexate in liver of rats pre-treated with BV, the present study found that concurrent treatment with BV had significantly decreased the methotrexate-induced elevation of serum TNF-α, ALT and AST levels, indicating the hepato-protective effect of BV, which might be explained by the reduction of elevated NF-κB expression in liver. This was further confirmed by histological examination of liver. These results are consistent with other studies that showed the potent hepato-protective effect of BV by inhibiting the secretion of pro-inflammatory cytokines, such as TNF-α, and decreasing the elevated serum amino-transferase enzymes in different models of induced hepatic injury, such as ethanol and actinomycin-D through the inhibition of deoxyribonucleic acid damage [61], [62] which may also interfere with the direct hepatotoxicity of methotrexate, which is believed to be through inhibition of RNA and DNA synthesis in the liver and increased hepatic stellate cell numbers [63].

## Conclusion

The present study demonstrated that the BV potentiates the anti-arthritic effects of methotrexate, possibly by reducing both NF-κB and TNF-α expression, and increasing methotrexate bioavailability in targeted sites. In addition, concurrent administration with BV provides a potent anti-nociceptive effect in the rat adjuvant arthritis model. Furthermore, BV mitigated the methotrexate induced hepatotoxicity mostly due to its inhibitory effect on serum TNF-α and tissue NF-κB. So, if similar effects occur in RA patients, the corporation of BV into methotrexate based therapy would offer a promising alternative modality in the treatment of RA, as it enhances the therapeutic potency and minimize the main associated adverse effects of methotrexate. Therefore, further study of the pharmacokinetic interaction between BV and methotrexate in diseased state is required, and the possibility of clinical application of such combination.
